# Online monitoring of the respiratory quotient reveals metabolic phases during microaerobic 2,3‐butanediol production with *Bacillus licheniformis*

**DOI:** 10.1002/elsc.201900121

**Published:** 2019-11-28

**Authors:** Benedikt Heyman, Hannah Tulke, Sastia Prama Putri, Eiichiro Fukusaki, Jochen Büchs

**Affiliations:** ^1^ AVT‐Biochemical Engineering RWTH Aachen University Aachen Germany; ^2^ Department of Biotechnology Graduate School of Engineering Osaka University Osaka Japan

**Keywords:** 2,3‐butanediol, *Bacillus licheniformis*, microaerobic, mixed acid fermentation, respiratory quotient

## Abstract

Microaerobic cultivation conditions are often beneficial for the biotechnological production of reduced metabolites like 2,3‐butanediol. However, due to oxygen limitation, process monitoring based on oxygen transfer rate, or dissolved oxygen measurement provides only limited information. In this study, online monitoring of the respiratory quotient is used to investigate the metabolic activity of *Bacillus licheniformis* DSM 8785 during mixed acid‐2,3‐butanediol production under microaerobic conditions. Thereby, the respiratory quotient provides valuable information about different metabolic phases. Based on partial reaction stoichiometries, the metabolic activity in each phase of the cultivation was revealed, explaining the course of the respiratory quotient. This provides profound information on the formation or consumption of glucose, 2,3‐butanediol, ethanol and lactate, both, in shake flasks and stirred tank reactor cultivations. Furthermore, the average respiratory quotient correlates with the oxygen availability during the cultivation. Carbon mass balancing revealed that this reflects the increased formation of reduced metabolites with increasing oxygen limitation. The results clearly demonstrate that the respiratory quotient is a valuable online signal to reveal and understand the metabolic activity during microaerobic cultivations. The approach of combining respiratory quotient monitoring with stoichiometric considerations can be applied to other organisms and processes to define suitable cultivation conditions to produce the desired product spectrum.

**Abbreviations**
CTR: carbon dioxide transfer rate
DOT: dissolved oxygen tension
OTR: oxygen transfer rate
OTR_max_: maximum oxygen transfer capacity
RQ: respiratory quotient

## 1 INTRODUCTION

In aerobic bioprocesses, the agitation and aeration demands for ensuring sufficient oxygen supply significantly contribute to the overall energy consumption 1. Consequently, cultivation processes with reduced oxygen transfer are economically attractive. Furthermore, so called microaerobic cultivation conditions are often beneficial for the biotechnological production of various products including amino acids, vitamins, polysaccharides, antibiotics, vaccines, enzymes, and alcohols 2. Microaerobic conditions are achieved, when the oxygen transfer from the gaseous to the aqueous phase is lower than the microorganisms’ oxygen demand. Consequently, the dissolved oxygen tension (DOT) is close to zero. Under these oxygen limited conditions, the intracellular NADH_2_/NAD balance cannot solely be maintained by oxidation of NADH_2_ in the respiratory chain. Consequently, the formation of reduced metabolites or organic acids is favored to regenerate NAD to maintain energy production via glycolysis 3.

Microbial based 2,3‐butanediol production is a much studied example for microaerobic cultivation processes. The compound has a wide range of applications as platform molecule in the chemical industry 4, 5. Therefore, biotechnological 2,3‐butanediol production is a promising approach to decrease the dependency of the chemical industry on fossil resources. 2,3‐Butanediol is produced in a mixed acid‐2,3‐butanediol fermentation pathway 6. Depending on the oxygen availability, different by‐products like acetoin, glycerol, ethanol, and organic acids are formed 7, 8, 9, 10. This leads to reduced product yields, as well as more challenging downstream processing 11, 12. Consequently, online monitoring of the production process to understand and control the underlying mechanisms of by‐product formation is an essential step towards commercially feasible 2,3‐butanediol production.

The limited availability of online process‐monitoring and ‐control possibilities is a general challenge in microaerobic cultivations. The DOT, which is often used as control parameter in aerobic cultivations, is close to zero (in the range of the K_m_‐value for oxygen) under microaerobic conditions. In this range the DOT cannot be monitored with DOT probes 13. Additionally, the oxygen transfer rate (OTR) is constant at the level of the maximum oxygen transfer capacity (OTR_max_). Therefore, many metabolic phenomena like diauxic effects, limitations and inhibitions that affect the course of the OTR in aerobic cultivations cannot be observed 14. In contrast, the respiratory quotient (RQ) has been successfully applied for process monitoring and control during microaerobic cultivations 15, 16, 17, 18, 19, 20. Furthermore, Zeng, et al. 3 used the RQ to control the oxygen supply for 2,3‐butanediol production and to monitor the viable biomass during the cultivation 21. The RQ is the quotient of carbon dioxide formation and oxygen consumption and provides additional information about the metabolic activity of the organism. Depending on the utilized carbon source, biomass formation, and the formation of overflow metabolites, different RQs are observed. Generally, an RQ of 1 results when substrate and product have the same degree of reduction. The RQ has values above 1 when the product is more reduced than the substrate and values below 1 in the opposite case. For a given reaction stoichiometry, a theoretical RQ can be calculated based on oxygen consumption and carbon dioxide formation.

**PRACTICAL APPLICATION**

The presented combination of online monitoring of the respiratory quotient (RQ) with stoichiometric analyses provides detailed information about the metabolic activity during microaerobic cultivations. Thereby, the same results were obtained in stirred tank reactor and shake flask cultivations. The discovered phases during the microaerobic cultivation of *Bacillus licheniformis* enhance the understanding of this specific organism. This can directly be applied to optimize cultivations of *B. licheniformis* by enhancing or reducing the formation of biomass, or specific products and by‐products like 2,3‐butanediol, glycerol, acetoin, ethanol, and organic acids. Furthermore, reaction stoichiometry and RQ are independent of the utilized microorganism. Consequently, the described methodologies can be applied to different microorganisms and products. Thereby, the information content of online monitoring during microaerobic cultivations can be increased. This helps to understand, characterize and optimize different cultivation processes without the need for excessive offline sampling.

Despite the above mentioned benefits, there is not much literature about RQ monitoring of microaerobic cultivations. Most of these studies either focused on process control and scale‐up strategies 3, 8, 15, 18 or investigated the formation of ethanol 17, 20, and 1,3‐propanediol 16, 19. Therefore, the aim of this study is to understand the metabolic activity in different cultivation phases of the mixed acid‐2,3‐butanediol production and to improve the explanatory power of online RQ monitoring. The highest reported 2,3‐butanediol concentrations with non‐pathogenic organisms were achieved with *Bacillus licheniformis* DSM 8785 22. Therefore, microaerobic cultivations of this organism were investigated with the aim to provide concepts and insights that can also be transferred and applied to different microorganisms. To date, the RQ during microaerobic cultivations has only been investigated in stirred tank reactor cultivations. In contrast, this study addresses both, shake flasks and stirred tank reactor cultivations. Thereby, the obtained results are independent of the utilized bioreactor system and scale. According to the measured RQ, partial reaction stoichiometries were set up for each metabolic phase. Thereby, the respective predominant metabolic activity was identified and confirmed by offline sampling. Finally, the average RQ was correlated to the OTR_max_ of different cultivations, reflecting the metabolic response to changing oxygen availability.

## 2 MATERIALS AND METHODS

### 2.1 Microorganism and culture medium

*Bacillus licheniformis* DSM 8785 was cultivated in batch mode in all experiments. The strain was obtained from Leibniz Institute DSMZ – German Collection of Microorganisms and Cell Cultures and stored in 150 g/L glycerol stocks at −80°C. The culture medium used for pre‐ and main‐culture was based on a medium described by Nakashimada, et al. 23. The medium contained 180 g/L glucose, 5 g/L yeast extract (Karl Roth GMBH, Karlsruhe, Germany), 5 g/L tryptone (Karl Roth GMBH, Karlsruhe, Germany), 7 g/L K_2_HPO_4_, 5.5 g/L KH_2_PO_4_, 1 g/L (NH_4_)_2_SO_4_, 0.25 g/L MgSO_4_·7H_2_O, 0.12 g/L Na_2_MoO_4_·2H_2_O, 0.021 g/L CaCl_2_·2H_2_O, 0.029 g/L Co(NO_3_)_2_·6H_2_O, 0.039 g/L (NH_4_)_2_Fe(SO_4_)_2_·6H_2_O, 0.002 g/L nicotinic acid, 0.0002 g/L Na_2_SeO_3_, 0.00005 g/L NiCl_2_·6H_2_O, 0.005 g/L MnCl_2_·4H_2_O, 0.001 g/L H_3_BO_3_, 0.0002 g/L AlK(SO_4_)_2_·12H_2_O, 0.00001 g/L CuCl_2_·2H_2_O, and 0.0055 g/L Na_2_EDTA·2H_2_O. In shake flask cultivations, 100 mM MES (2‐[*N*‐morpholino]ethanesulfonic acid) buffer was added for pH control. In stirred tank reactor cultivations, pH values above 5.5 were maintained by automated addition of 2 M NaOH. In both reactor types the initial pH was adjusted to 6.5 by addition of NaOH.

### 2.2 Shake flask cultivations

250 mL unbaffled shake flasks were placed on an orbital shaker (Climo shaker ISF1‐X, Adolf Kühner AG, Birsfelden, Switzerland) operated at a shaking diameter of 50 mm at 37°C. OTR, carbon dioxide transfer rate (CTR), and RQ were monitored online with an in‐house build Respiration Activity Monitoring System (RAMOS) 24 in duplicates. The measurement principle is explained in detail by Anderlei, et al. 24 and is based on a continuously repeated cycle of actively gassed and ungassed phases. During the ungassed measurement phases, the changes of oxygen and carbon dioxide partial pressures over time are recorded in the headspace of the shake flask. One OTR and CTR value is calculated during each measurement phase, resulting in a measurement interval of 0.5 h. Commercial versions can be purchased from HiTec Zang GMBH (Herzogenrath, Germany) or Adolf Kühner AG (Birsfelden, Switzerland). Offline samples were taken from parallel cultivations in conventional shake flasks under identical cultivation conditions. For each sampling point, one flask was removed for sampling and discarded afterwards. Consequently, each dataset is derived from multiple parallel cultivations as visualized in Supporting Information Figure S1. Thereby, the time course of offline samples and the overlapping information between offline samples and online monitored respiration activity show the consistency and reproducibility of the obtained results. Pre‐cultures were cultivated at 350 rpm after inoculation of 20 mL medium per flask with 20 µL of a glycerol stock. The cells were harvested in the exponential growth phase (determined from the online measured OTR) and a master mix was inoculated to an initial optical density (OD_600_) of 0.1. Each flask of the main culture was then filled from the master mix. Different filling volumes were used as specified in the respective figure legends. At the beginning of the cultivation, a shaking frequency of 350 rpm was applied to enable oxygen unlimited growth conditions. At a defined OTR of 20 mmol/L/h the shaking frequency was reduced to 100 rpm to initiate oxygen limitation.

### 2.3 Stirred tank reactor cultivations

Stirred tank reactor cultivations were performed in a 2 L Autoclavable Bioreactor (Applikon Biotechnology, Foster City, USA) with three baffles and two Rushton Turbines (six blades, 45 mm stirrer diameter) at 1.5 L filling volume. The reactor was equipped with sensors for pH and DOT measurement (AppliSens pH and pO2 sensor, Applikon Biotechnology, Foster City, USA). OTR, CTR, and RQ were derived from measurements of carbon dioxide and oxygen concentrations in the exhaust gas at a measurement interval of 1 min without smoothing or filtering (X‐STREAM exhaust gas analyzer, Emerson Process Management GmbH, Wessling, Germany). The reactor was aerated via an L‐type gas sparger at the reactor bottom with pressurized air at an aeration rate of 0.5 L/min (0.33 vvm). Plurafac LF 1300 anti‐foaming agent (BASF, Ludwigshafen, Germany) was added before inoculation (2 mL) and when foaming was observed during the cultivation (1 mL). The reactor was inoculated with 20 mL of an overnight pre‐culture (pre‐culture conditions as defined in Section 2.2). At the beginning of the cultivation, a high agitation rate (1000 rpm) was applied to enable oxygen unlimited growth conditions. At a defined OTR of 20 mmol/L/h, the agitation rate was reduced to 500 rpm to initiate oxygen limitation. The cultivation temperature was set to 37°C and regulated using a Pt‐100 sensor (Applikon Biotechnology, Foster City, USA) and the integrated cooling jacket.

### 2.4 Offline sampling

**Figure 1:**
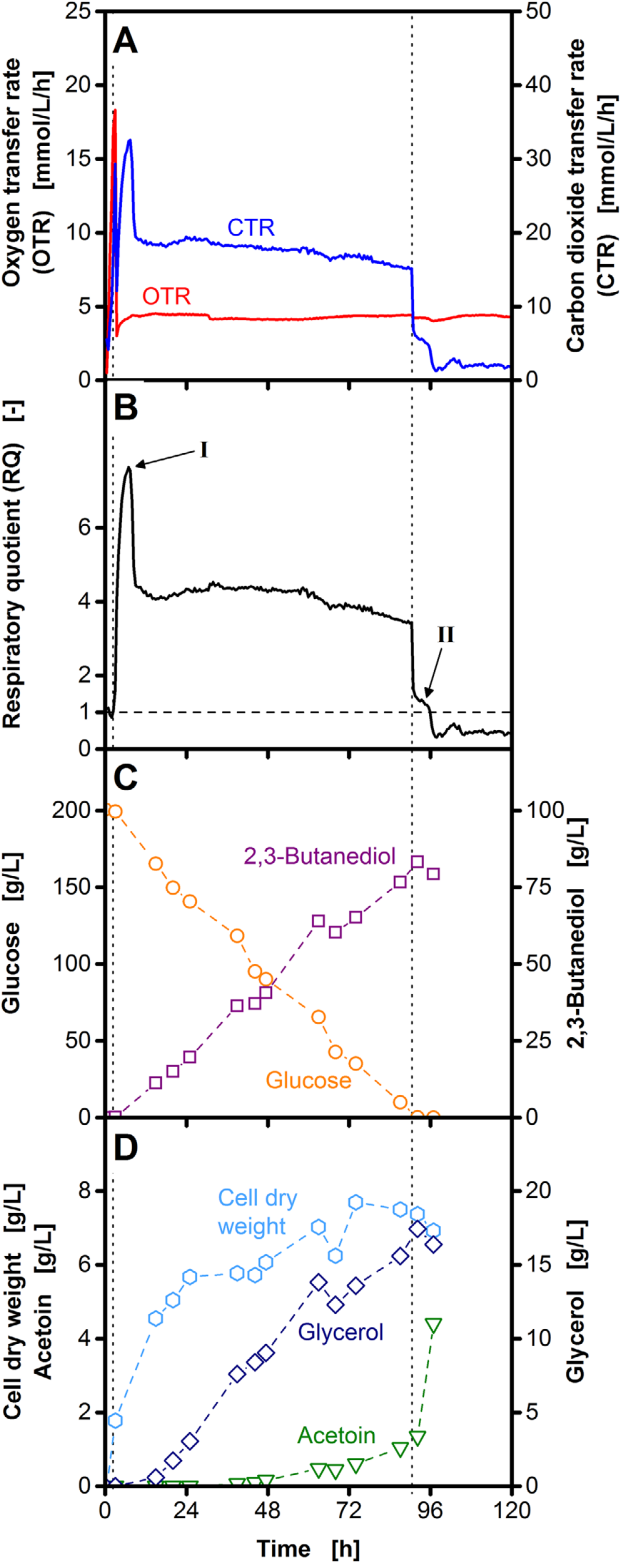
Shake flask cultivation of *Bacillus licheniformis* DSM 8785 under microaerobic conditions. The courses of oxygen (OTR) and carbon dioxide transfer rates (CTR) (A), the respiratory quotient (RQ) (B) as well as glucose, 2,3‐butanediol (C), acetoin, glycerol, and cell dry weight (D) are shown. The shaking frequency was reduced from 350 to 100 rpm after 3 h (left vertical dotted line). As visualized by the vertical dotted lines, an oxygen unlimited growth phase is followed by the 2,3‐butanediol production phase and subsequent 2,3‐butanediol consumption. The peak (I) and the shoulder (II) in the RQ course (B) are investigated in this study. Offline data are derived from an individual shake flask at each time point. Cultivation conditions: 250 mL unbaffled shake flasks, temperature: 37°C, filling volume: 40 mL, shaking frequency: 350/100 rpm, shaking diameter: 50 mm

For shake flask cultivations, evaporation of water was determined based on the remaining liquid volume. An evaporation rate of 0.04 mL/h was observed (e.g. 10% volume loss due to evaporation after 100 h in Figure 1). Thus, all concentrations were corrected with respect to evaporation to prevent overestimation. In stirred tank reactor cultivations, an exhaust gas cooler was installed to minimize evaporation, which was not further accounted for. For each sample, two 2 mL reaction tubes were each filled with 2 mL culture broth and centrifuged (5 min at 17 968 g) to separate the cells. The cell dry weight was determined gravimetrically in pre‐dried and ‐weighted reaction tubes after two washing steps with deionized water. The obtained pellet was dried for at least 48 h at 60°C. Metabolite concentrations were determined via HPLC in the filtered supernatant (0.2 µm). The supernatant was suitably diluted with deionized water to obtain metabolite concentrations between 1 and 10 g/L. A Dionex UltiMate 3000 HPLC system (Dionex, Sunnyvale, USA) was equipped with an organic acid‐resin (250 · 8 mm, CS‐Chromatographie, Langerwehe, Germany) and operated at 70°C. A Shodex RI‐101 (Showa Denko, Munich, Germany) detector was used. A total of 2.5 mM sulfuric acid was used as mobile phase at a flow rate of 0.5 mL/min. *B. licheniformis* produces a mixture of meso‐ and d‐2,3‐butanediol 25, 26. With the described chromatographic method, these stereoisomers are detected separately. All given concentrations refer to the sum of both 2,3‐butanediol stereoisomers. The pH was determined directly after sampling using a CyberScan pH 510 (Eutech Instruments, Landsmeer, The Netherlands) pH meter.

### 2.5 Calculations and stoichiometry

The theoretical RQ during different phases of the cultivation was calculated based on partial reaction stoichiometries. In the metabolic network, these reactions are coupled and do not occur separately. In the course of the cultivation, changing combinations of all partial reactions result in the overall metabolic activity. Stoichiometric coefficients of carbon dioxide formation ($v_{CO_{2}}$) and oxygen consumption ($v_{O_{2}}$) are needed to calculate the theoretical RQ according to Eq. (1).

(1) $$RQ=\frac{\nu_{CO_{2}}}{\nu_{O_{2}}}$$

Eq. (2) shows the stoichiometry of 2,3‐butanediol production from glucose:

(2) $$C_{6}H_{12}O_{6}+\mathrm{NAD}\to C_{4}H_{10}O_{2}+2\;CO_{2}+{\mathrm{NADH}}_{2}$$

The produced NADH_2_ can be oxidized in the respiratory chain to generate ATP (not shown in Eq. (3)) and to regenerate NAD according to Eq. (3):

(3) $$O_{2}+2\;{\mathrm{NADH}}_{2}\to 2\;H_{2}O+2\;\mathrm{NAD}$$

Eq. (4) results from the combination of Eq. (2) and (3) and allows easy determination of $v_{CO_{2}}$ and $v_{O_{2}}$ to calculate the RQ according to Eq. (1):

(4) $$C_{6}H_{12}O_{6}+0.5\;O_{2}\to C_{4}H_{10}O_{2}+2\;CO_{2}+H_{2}O$$

Eq. (5), (6), (7) were derived from similar considerations to determine $v_{CO_{2}}$ and $v_{O_{2}}$ for the respective partial reaction stoichiometries.

To setup the stoichiometry of growth on glucose (Eq. (5)), a biomass composition for *B. licheniformis* of C_1_H_1.6_O_0.4_N_0.2_ was assumed based on biomass compositions of *B. subtilis* that were determined by Dauner, et al. 27 in N‐ and P‐limited chemostat experiments.

(5) $$\begin{array}{ccc} & & C_{6}H_{12}O_{6}+0.75\;O_{2}+{\mathrm{NH}}_{3}\to 5\;C_{1}H_{1.6}O_{0.4}N_{0.2}\\ & & \;+\;CO_{2}+3.5\;H_{2}O \end{array}$$

Under anaerobic conditions, ethanol is formed from glucose according to Eq. (6):

(6) $$C_{6}H_{12}O_{6}\to 2\;C_{2}H_{6}O+2\;CO_{2}$$

Lactate combustion follows the stoichiometry shown in Eq. (7):

(7) $$C_{3}H_{6}O_{3}+3\;O_{2}\to 3\;CO_{2}+3\;H_{2}O$$

The conversion of 2,3‐butanediol to acetoin after glucose depletion is shown in Eq. (8):

(8) $$C_{4}H_{10}O_{2}+\mathrm{NAD}\to C_{4}H_{8}O_{2}+{\mathrm{NADH}}_{2}$$

Glycerol is formed from glucose according to Eq. (9):

(9) $$C_{6}H_{12}O_{6}+2\;{\mathrm{NADH}}_{2}\to 2\;C_{3}H_{8}O_{3}+2\;\mathrm{NAD}$$

Eq. (8) and (9) do not include carbon dioxide formation. Thus, the RQ cannot be determined according to Eq. (1). However, glycerol formation (Eq. (9)) is accompanied by the conversion of NADH_2_ to NAD. Consequently, less NADH_2_ is oxidized via the respiratory chain (Eq. (3)) and the RQ increases. In contrast, conversion of 2,3‐butanediol to acetoin (Eq. (8)) results in a decreased RQ, as more NADH_2_ can be oxidized without carbon dioxide formation.

Based on Eq. (1), (2), (3), (4), (5), (6), (7), (8), (9), theoretical RQs were calculated for specific phases during the cultivation based on partial reaction stoichiometries. Additionally, average RQs for cultivations with different OTR_max_ were calculated. Based on the respective carbon mass balances, the produced CO_2_ was calculated considering all metabolites shown in Eq. (10). Thereby, only glucose (180 g/L) was considered as carbon source, while the carbon released from undefined components like yeast extract and tryptone (5 g/L each) could not be measured and was thus neglected. The carbon content (about 40%) in glucose, yeast extract, and tryptone is very similar 28, 29. Consequently, about 95% of the total carbon originates from glucose, resulting in comparable courses of OTR, CTR, and RQ with and without yeast extract and tryptone addition (Supporting Information Figure S5).

(10) $$\begin{array}{ccc} & & \nu_{Glu}Glucose+\nu_{O_{2}}O_{2}+\nu_{NH_{3}}NH_{3}\\ & & \;\to \nu_{But}2,3Butanediol+\nu_{Aceto}Acetoin+\nu_{Gly}Glycerol\\ & & \;+\;\nu_{Eth}Ethanol+\nu_{Aceta}Acetate+\nu_{Lac}Lactate\\ & & \;+\;\nu_{Suc}Succinate+\nu_{CDW}Cell\;dry\;weight\\ & & \;+\;\nu_{CO_{2}}CO_{2}+\nu_{H_{2}O}H_{2}O \end{array}$$

This calculation was performed for the 2,3‐butanediol production phase between the initiation of oxygen limited conditions and glucose depletion as depicted in Supporting Information Figure S2B. Oxygen limited conditions are induced by reduction of the shaking frequency. Glucose depletion can be detected online, as it results in a steep decrease of the RQ. The stoichiometric coefficients ν_i_ were determined from offline samples. The product concentrations upon glucose depletion were determined via linear interpolation as described and validated in a previous publication 10. To minimize the experimental error, also the glucose concentration at the initiation of the oxygen limitation was calculated from the glucose consumption rate. To do so, the glucose consumption rate was determined via linear regression from all samples taken during the 2,3‐butanediol production phase. Based on the obtained linear equation, the glucose concentration at the initiation of the 2,3‐butanediol production phase was calculated. The consumed oxygen during the 2,3‐butanediol production phase was calculated by integrating the measured OTR.

## 3 RESULTS

In this work, cultivations were performed in unbaffled 250 mL shake flasks and a 2 L stirred tank reactor. All shake flask cultivations were performed in at least seven shake flasks in parallel and the respiration activity was monitored online with the Respiration Activity Monitoring System (RAMOS) 24.

### 3.1 Cultivation of *B. licheniformis*

The cultivation of *B. licheniformis* under microaerobic conditions follows three distinct phases (Figure 1). An unlimited growth phase (until the first dotted line) is followed by the 2,3‐butanediol production phase (between the dotted lines) and finally 2,3‐butanediol is converted to acetoin (after the second dotted line). Figure 1A depicts the courses of oxygen transfer rate (OTR) and carbon dioxide transfer rate (CTR) in 250 mL shake flasks. A two‐stage cultivation profile was applied in all cultivations to enable defined microaerobic cultivation conditions as described in detail in a previous publication 10. At the beginning of the cultivation, a shaking frequency of 350 rpm results in oxygen unlimited growth as shown by the steep increase of the OTR. When the OTR reached values around 20 mmol/L/h, the shaking frequency was decreased to 100 rpm. Thereby, oxygen limitation was initiated, resulting in an OTR plateau at the level of the maximum oxygen transfer capacity (OTR_max_; cf. Supporting Information Figure S2A) of 5 mmol/L/h. The CTR peaks at 30 mmol/L/h at the beginning of the oxygen limited 2,3‐butanediol production phase and remains at 20 mmol/L/h until 90 h. Afterwards, a sharp decrease of the CTR to a shoulder at 6 mmol/L/h is followed by a second sharp decrease to 2 mmol/L/h at 96 h. Figure 1B shows the course of the RQ. A theoretical RQ can be calculated for a given reaction stoichiometry based on oxygen consumption and carbon dioxide formation (Eq. (1)). Thus, monitoring of the RQ can be combined with stoichiometric considerations to understand the metabolic activity during the cultivation. During the oxygen unlimited phase in the first 3 h of the cultivation, the RQ is around 1 indicating aerobic growth on glucose. Due to the high lipid content in the membrane, biomass is more reduced than glucose, resulting in a calculated RQ of 1.3 during biomass formation (Eq. (5)). However, this calculation does not include maintenance or other energy requiring processes. Thus, a slightly lower RQ can be expected, when a combination of growth and glucose combustion is assumed during the first 3 h of cultivation (Figure 1B). Afterwards, the course of the RQ resembles the course of the CTR, as the OTR stays constant due to oxygen limitation. The general course of the RQ can be explained by the courses of glucose, 2,3‐butanediol (Figure 1C), glycerol, and acetoin (Figure 1D). The RQ of 4 between 9 and 90 h mainly results from glucose consumption and 2,3‐butanediol production, as the calculated RQ for this reaction is 4 (Eq. (4)). Additionally, glycerol and biomass are formed at that time (Figure 1D). Compared to 2,3‐butanediol production, biomass formation reduces the RQ (Eq. (4) and (5)), whereas glycerol formation increases the RQ (Eq. (9)). Upon glucose depletion, 2,3‐butanediol is consumed and converted to acetoin (clearly visible in Supporting Information Figure S3). This conversion provides protons that can be oxidized in the respiratory chain (Eq. (8)) resulting in RQ values below 1. The above mentioned RQ course has been investigated and discussed in detail in a previous publication 10. However, the peak after 7 h and the shoulder between 90 and 96 h (highlighted as I and II in Figure 1B) cannot be explained by the above‐mentioned mechanisms and were investigated in this study.

For a comprehensive investigation of these phenomena, shake flask and stirred tank reactor cultivations were combined. Thereby, the benefits of both systems can be utilized. Shake flasks provide high experimental throughput and parallelization, whereas stirred tank reactors provide high reaction volumes and a technical setup that is closer to industrial production processes. The scalability of the investigated 2,3‐butanediol production process between shake flasks and stirred tank reactor was already demonstrated in a previous publication 10 (Supporting Information Figure S3). However, different oxygen transfer mechanisms are relevant in the different bioreactor systems. In unbaffled shake flasks, oxygen transfer from the gas to the liquid phase takes place at the liquid surface. The shape of the rotating bulk liquid in shake flasks only depends on the operation conditions and not on the physiochemical properties of the culture liquid. Thus, the mass transfer area is very defined and constant 30, 31. In contrast, oxygen transfer in stirred tank reactors occurs at the interface between gas bubbles and liquid. Thus, complex phenomena like bubble dispersion and coalescence or foaming affect the oxygen transfer 32, 33, 34, 35, 36. Consequently, combination of both systems guarantees that biological effects can be investigated under microaerobic conditions, independent of bioreactor specific oxygen transfer phenomena.

### 3.2 Investigation of the RQ peak

The metabolic causes for the RQ peak at the beginning of the 2,3‐butanediol production phase (marked as I in Figure 1B) were investigated. As described above, the RQ provides information on the degree of reduction of the utilized carbon source and the produced metabolites. Consumption of glucose and formation of 2,3‐butanediol results in RQs around 4 (Eq. (4)). The RQ peak can have two different metabolic causes, either the production of a by‐product that is more reduced than 2,3‐butanediol, or the consumption of a substrate that is more oxidized than glucose.

As the OTR is constant during the period of the RQ peak, it originates from a CTR peak (cf. Figure 1A). Thus, also non‐metabolic effects that cause additional carbon dioxide release could be an explanation for the observed peak. When the pH decreases during the cultivation in the range of the pK_a_ value of the bicarbonate buffer (6.1), previously dissolved carbon dioxide can be released from the culture broth. This carbon dioxide will then be detected in the gas phase and result in an overestimation of the CTR. To exclude any effects related to changing pH values, the pH can be controlled during the cultivation. However, pH control during the cultivation of *Bacillus licheniformis* DSM 8785 impaired 2,3‐butanediol production (Supporting Information Figure S4). Furthermore, pH control resulted in oscillating courses of OTR and RQ. This oscillation might be caused by switching metabolic activity as the mixed acid‐2,3‐butanediol fermentation is, amongst others, regulated by the course of the pH 7. Therefore, a forced constant pH might interfere with this regulation. A comparable behavior of *B. licheniformis* has been observed in pH controlled fed‐batch cultivations in the literature 37, 38. Even though these authors did not show OTR data, the agitation rate fluctuated during phases of controlled and constant DOT. This indicates similar switching metabolic activity as observed from the oscillating OTR at constant agitation observed in this study (Supporting Information Figure S4). As pH control completely changed the cultivation characteristics and impaired 2,3‐butanediol production, pH control was not further considered in this study.

**Figure 2:**
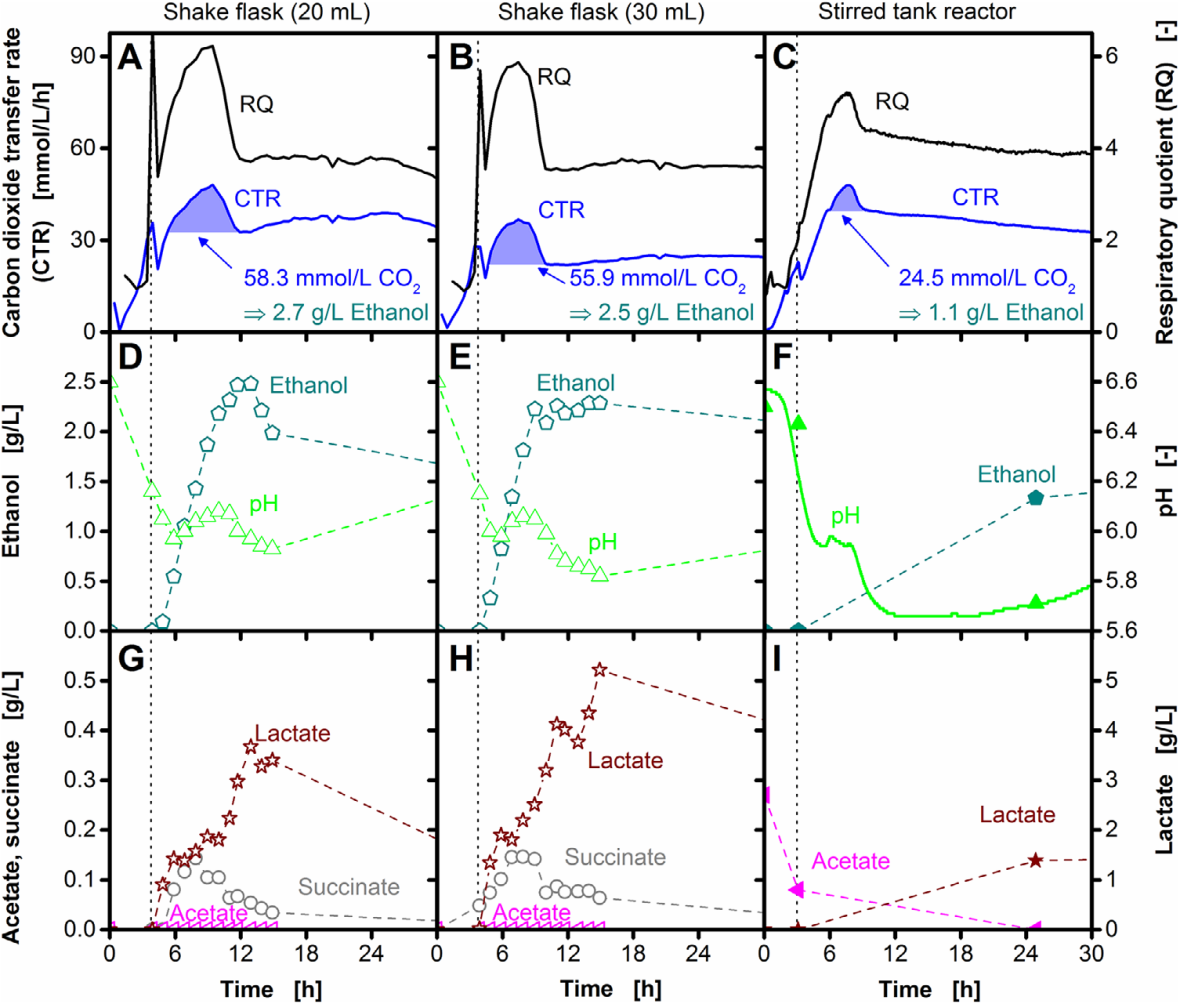
Investigation of the peak in the respiratory quotient. The formation of the peak (marked as I in Figure 1B) is investigated in shake flask (A, B, D, E, G, H) and stirred tank reactor (C, F, I) cultivations. Carbon dioxide transfer rates (CTR) and respiratory quotients (RQ) (A–C), ethanol concentrations and pH values (D–F) as well as organic acid concentrations (G–I) are shown. In the stirred tank reactor, the pH was measured online (line) and offline (symbols). According to Eq. (6), ethanol formation under microaerobic conditions results in equimolar carbon dioxide formation (blue colored areas under the CTR peak in (A–C). The shaking frequency and the agitation rate were reduced from 350 to 100 and from 1000 to 500 rpm after 3 and 4 h, respectively (vertical dotted lines). For shake flask experiments, offline data are derived from an individual shake flask at each time point. For the stirred tank reactor experiment, all samples were taken from one reactor. Cultivation conditions: Temperature: 37°C; shake flask cultivation: 250 mL unbaffled shake flasks, shaking frequency: 350/100 rpm, shaking diameter: 50 mm; stirred tank reactor cultivation: 2 L stirred tank reactor, filling volume: 1.5 L, aeration rate: 0.5 L/min (0.33 vvm), agitation rate: 1000/500 rpm

To investigate the cause of the RQ peak, two shake flask cultivations with different filling volumes and a stirred tank reactor cultivation are compared in Figure 2. The filling volume of shake flask cultivations determines the OTR_max_ (Supporting Information Figure S2) and, thus, severely influences the formation of by‐products 8, 10. Figure 2A–C depicts the CTRs and RQs during the first 30 h of cultivation, forming the previously observed peaks. The short fluctuation of CTR and RQ at 3 and 4 h is caused by the respective reduction of the shaking frequency and does not occur in experiments without reduction of the shaking frequency (Supporting Information Figure S5). For all cultivation conditions, the pH decreases from 6.6 to 6 during the first hours of cultivation before the RQ peak occurs (Figure 2D–F). During the RQ peak, the pH slightly increases and decreases after the peak. Consequently, the course of the pH cannot explain the RQ peak. Equilibrium changes of the bicarbonate buffer only cause carbon dioxide release from the culture broth at decreasing pH values. Thus, with the observed pH profile the measured CTR would rather be decreased than increased. Apart from equilibrium changes of the bicarbonate buffer, the specific aeration rate has a predominant influence on the carbon dioxide release from the culture broth 39. As the specific aeration rate is not changed during the cultivation, this does not explain the formation of an RQ peak. Under the given cultivation conditions, mixing times in the range of 10 s can be expected 40. As the RQ peak lasts for at least 5 h, delayed carbon dioxide release due to limited mixing at the low shaking frequency of 100 rpm can also be excluded.

Ethanol, a highly reduced by‐product, is formed during the RQ peak (Figure 2D–F). Under microaerobic cultivation conditions, ethanol formation results in equimolar carbon dioxide formation (Eq. (6)). To evaluate if this carbon dioxide formation from ethanol production causes the RQ peak, the observed carbon dioxide formation can be compared to the theoretically expected carbon dioxide formation. The excess carbon dioxide formation during the period of the peak time (blue colored areas in Figure 2A–C) was calculated to 58, 56, and 25 mmol/L/h, which correlates to the formation of 2.7, 2.5, and 1.1 g/L ethanol for the shake flasks with 20 and 30 mL and the stirred tank reactor cultivation, respectively. This is in good agreement with the offline measured ethanol concentrations in the shake flask experiments (ca. 2.5 g/L each; Figure 2D and E). In the stirred tank reactor cultivation, the exact ethanol concentration during the time of the RQ peak cannot be compared due to missing offline samples at this exact time. However, the measured ethanol concentration is also in the expected range (Figure 2F). The good correlation between the theoretically expected and observed carbon dioxide formation indicates that ethanol formation is the sole reason for the peak in the RQ at about 7 h.

Under microaerobic conditions, 2,3‐butanediol production is initiated as response to previous organic acid formation 7. As some organic acids (e.g. succinate) are more oxidized than glucose, organic acid formation during the period of the RQ peak was investigated. Concentrations of succinate, acetate, and lactate are shown in Figure 2G–I. In addition to these organic acids, formate formation was reported in the literature 7, which could not be detected in the conducted experiments. Only consumption of acetate (probably carried over from the pre‐culture) could be observed during the RQ peak in the stirred tank reactor cultivation. No organic acid consumption was observed in the shake flask experiments at this time. As acetate has the same degree of reduction as glucose, acetate formation cannot cause the RQ peak. Up to 0.2 g/L succinate, which is more oxidized than glucose, was formed during the period of the RQ peak. As succinate production leads to a decreased RQ, this is also not the cause of the RQ peak.

Neither pH‐changes (Figure 2D–F), nor organic acid consumption (Figure 2G–I) can explain the RQ peak. Formation of biomass from glucose results in an RQ of 1.3 (Eq. (5)). However, in addition to glucose, also yeast extract and tryptone are present and could be used for biomass formation. The composition of these supplements is not well defined and the RQ peak could theoretically be a result of biomass formation from an unknown oxidized ingredient. However, the RQ peak also occurs when *B. licheniformis* is cultivated in a chemically defined mineral medium (Supporting Information Figure S5). This confirms that the RQ peak is not caused by any undefined and unknown compound in the medium, but is a result of ethanol formation (Figure 2D–F).

### 3.3 Investigation of the RQ shoulder

**Figure 3:**
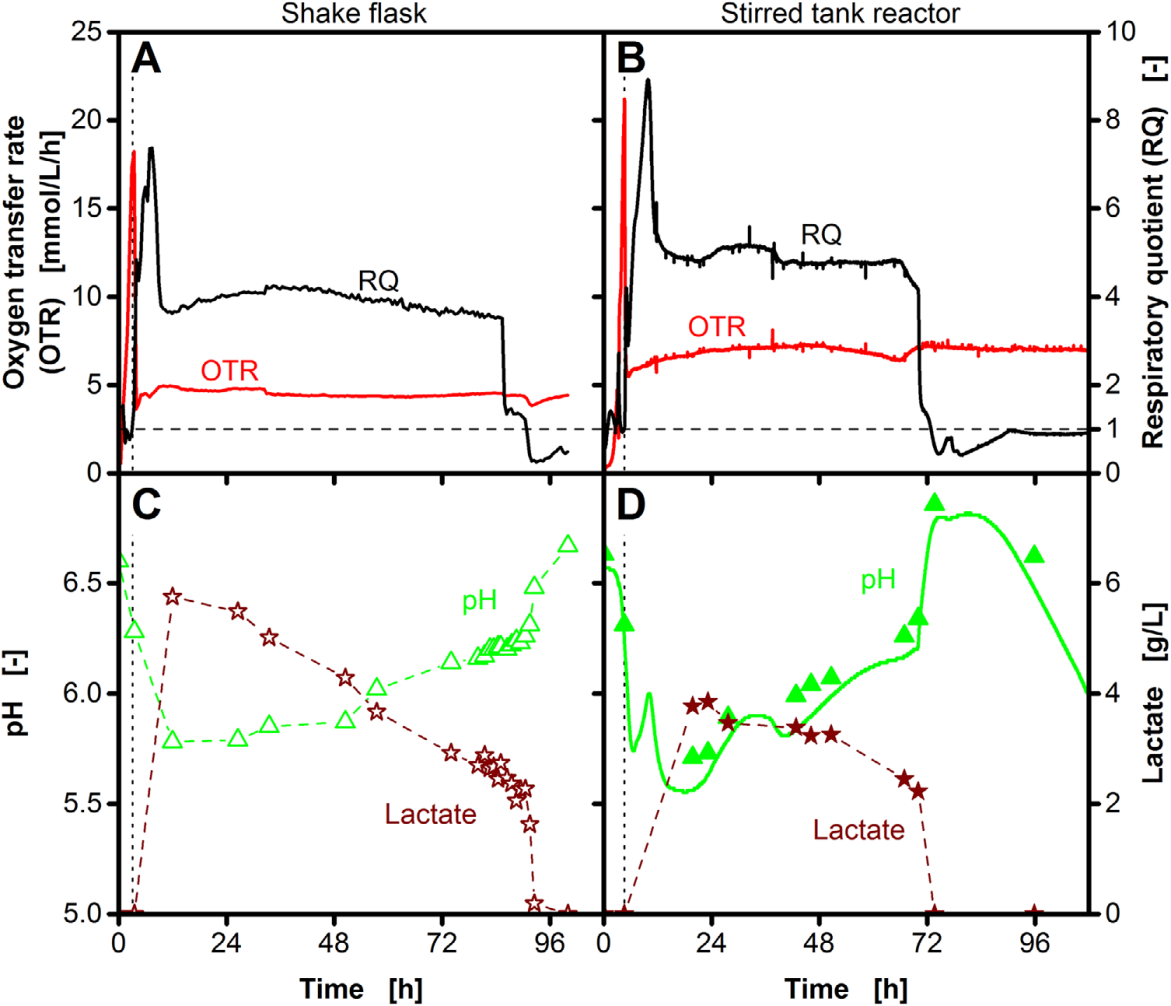
Investigation of the shoulder in the respiratory quotient. The formation of the shoulder (marked as II in Figure 1B) is investigated in shake flask (A, C) and stirred tank reactor (B, D) cultivations. Oxygen transfer rates (OTR) and respiratory quotients (RQ) (A, B) and the pH and lactate concentrations (C, D) are shown. In the stirred tank reactor, the pH was measured online (line) and offline (symbols). The shaking frequency and the agitation rate were reduced from 350 to 100 and from 1000 to 500 rpm after 4.5 and 3.5 h, respectively (vertical dotted lines). For the shake flask experiment, offline data are derived from an individual shake flask at each time point. For the stirred tank reactor experiment, all samples were taken from one reactor. Cultivation conditions: temperature: 37°C; shake flask cultivation: 250 mL unbaffled shake flasks, filling volume: 40 mL, shaking frequency: 350/100 rpm, shaking diameter: 50 mm; stirred tank reactor cultivation: 2 L stirred tank reactor, filling volume: 1.5 L, aeration rate: 0.5 L/min (0.33 vvm), agitation rate: 1000/500 rpm

The sharp drop of the RQ at the end of the 2,3‐butanediol production phase is caused by glucose depletion (cf. Figure 1). The formation of a shoulder (marked as II in Figure 1B) during this drop is only observed for strong oxygen limitations at low OTR_max_ (Supporting Information Figure S2). With increasing OTR_max_, the shoulder becomes smaller and finally disappears completely. To investigate the RQ shoulder, a shake flask and a stirred tank reactor cultivation are compared in Figure 3. The shake flask cultivation (Figure 3A) has a slightly lower OTR_max_ resulting in a longer RQ shoulder compared to the stirred tank reactor cultivation (Figure 3B). The formation of the RQ shoulder is accompanied by a rapidly decreasing lactate concentration, resulting in a simultaneous increase of the pH (Figure 3C and D). As specified by Eq. (7), combustion of lactate results in a theoretical RQ of 1. The measured RQ during the shoulder is slightly above 1 (at 90 and 72 h in Figure 3A and B, respectively). However, also other factors like the consumption of non‐detected by‐products most likely have an effect on the level of the RQ during this time. Therefore, lactate combustion is not the only, but the major factor causing the shoulder in the RQ.

### 3.4 Correlation between average RQ and OTR_max_

**Figure 4:**
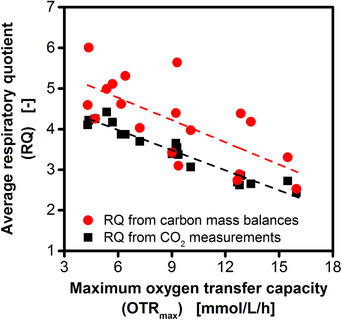
Correlation between average respiratory quotient and maximum oxygen transfer capacity (OTR_max_). Average respiratory quotients (RQ) from 19 individual shake flask cultivations at varied maximum oxygen transfer capacities (OTR_max_) are shown. The OTR_max_ was derived from the course of the OTR (cf. Supporting Information Figure S2). To calculate the RQ, carbon dioxide formation during 2,3‐butanediol production was either measured online, or derived from carbon balances of offline samples (cf. Supporting Information Figure S2, Eq. (10)). The metabolite and cell dry weight formation during the 2,3‐butanediol production phase is summarized in Supporting Information Tables S1 and S2. The consumed oxygen for RQ calculations was derived from measured oxygen transfer rates. Cultivation conditions: 250 mL unbaffled shake flasks, temperature: 37°C, shaking frequency: 350/100 rpm, shaking diameter: 50 mm

In the previously described results, the RQ was used to understand the metabolic activity during specific phases of the cultivation. In addition to these learnings, the average RQ during a longer cultivation period provides information on the overall stoichiometry of the cultivation. Therefore, the average RQ during the 2,3‐butanediol production phase was determined. In shake flasks, the 2,3‐butanediol production phase is initiated by the reduction of the shaking frequency to induce oxygen limitation and ends when the RQ sharply drops upon glucose depletion (visualized in Supporting Information Figure S2). The average RQ was determined by two different approaches. For the conventional determination, the online measured carbon dioxide formation and oxygen consumption are divided according to Eq. (1). Additionally, carbon mass balancing (Eq. (10); Supporting Information Tables S1 and S2) based on measured metabolite and cell dry weight concentrations was used to calculate the carbon dioxide formation, which was then divided by the online measured oxygen consumption. Figure 4 shows the correlation between the average RQ and the OTR_max_ for 19 individual experiments. The OTR_max_ is one of the most influential parameters during microaerobic cultivations and strongly determines the product and by‐product spectrum 8, 10. With increasing OTR_max_, the average RQ from online carbon dioxide measurement (black symbols) decreases linearly from 4 to 2.5. As discussed in Section 4, the decline of the average RQ with increasing OTR_max_ is well expected. More importantly, also the average RQ from carbon mass balances (red symbols) decreases from 5 to 3 with increasing OTR_max_. Even though generally higher offline RQs were observed, the overall trend matches the measured results. This confirms that the dependency of the average RQ of the OTR_max_ reflects the adaption of the metabolism to changing oxygen availability. With decreasing OTR_max_, the amount of energy that can be obtained by oxidation in the respiratory chain decreases 3. Consequently, more energy has to be generated via glycolysis. To regenerate NADH_2_ and to maintain glycolysis, more reduced overflow metabolites have to be formed (Supporting Information Tables S1 and S2) resulting in an increased average RQ.

## 4 DISCUSSION

Based on the course of the RQ, the cultivation can be divided into different metabolic phases. With the initiation of oxygen limited conditions, the 2,3‐butanediol production phase starts (Figure 1). At the beginning of this phase, additional ethanol formation results in a RQ peak (cf. Figure 2B). Afterwards, a stable RQ plateau is observed until glucose is depleted. At that point, the RQ rapidly drops to values below 1. In cultivations with low OTR_max_, utilization of the previously formed lactate results in a formation of a shoulder during this drop (cf. Figure 3). A very similar course of the RQ was observed for the cultivation of *B. vallismortis* 41. Furthermore, Zeng, et al. 3 concluded that increased RQs can be expected upon ethanol formation. However, to the authors’ knowledge, the metabolic reasons for the observed peak and shoulder have not yet been described in the literature.

The average RQ during the 2,3‐butanediol production phase correlates with the OTR_max_ during the respective cultivation (Figure 4). Rebecchi, et al. 8 also observed decreasing RQs for cultivations with increasing OTR_max_. However, it is not clear if this refers to average RQs or to RQs measured at a specific time during the cultivation. Additionally, Zeng, et al. 21 observed decreasing RQs at increased oxygen uptake rates during 2,3‐butanediol production in continuous cultivations of *E. aerogenes*. Calculation of the average RQ based on carbon mass balances from offline samples yielded generally higher RQs and shows a higher variation compared to RQs derived from online carbon dioxide measurements. The cultivations were performed in a complex medium in the presence of undefined components like yeast extract and tryptone. Their contribution to the carbon balance was neglected. Consequently, exact calculation of the RQ could not be expected. Still the overall trend matches the measured RQs. This clearly indicates that the observed correlation reflects the organism's metabolic response to varied oxygen availability as explained in Section 3.4.

RQ control can be used to manipulate and optimize the product spectrum. In a previous publication, the average RQ was adjusted by variations of the filling volume in shake flask experiments (Supporting Information Figure S6) 10. Thereby, the concentrations and formation rates of major products could be altered to select suitable cultivation conditions. Moreover, dynamic RQ control strategies have already been applied to optimize ethanol 15, 17 and 2,3‐butanediol 3 production under microaerobic conditions. Additionally, the results presented in this study indicate that dynamic RQ control strategies could be beneficial to optimize 2,3‐butanediol production by selection of suitable RQs for each cultivation phase.

Two approaches were presented to utilize online monitoring of the RQ to gain deeper insights into the metabolic activity of a culture under microaerobic cultivation conditions. Based on the course of the RQ, different metabolic phases during the cultivation were identified. Combined with partial reaction stoichiometries, the predominant metabolic activities in these phases were revealed. Additionally, the average RQs in the 2,3‐butanediol production phase of 19 individual cultivations at varied OTR_max_ reflect the organism's metabolic adaption to varied oxygen availability. The combination of online RQ monitoring with stoichiometric considerations is generally applicable to other microorganism and products. Thus, the presented approaches increase the potential of online process monitoring and characterization tools for microaerobic cultivations.

## CONFLICT OF INTEREST

The authors have declared no conflict of interest.

## Supporting information

Supporting Information
