# Supplementary material for: Online monitoring of the respiratory quotient reveals metabolic phases during microaerobic 2,3‐butanediol production with Bacillus licheniformis
Source: Eng Life Sci. 2019 Nov 28;20(3-4):133–44. doi: 10.1002/elsc.201900121 (PMC7447875; doi:10.1002/elsc.201900121)
Supplement: Supplementary file 1 — Supporting Information [file ELSC-20-133-s001.pdf]

Table S1: Metabolite and cell dry weight formation during the 2,3-butanediol production phase. This table provides additional information to Fig. 4. The metabolite formation during the 2,3-butanediol production phase (between t_1 and t_2 in Fig. S2) is shown for 19 individual shake flask cultivations with different maximum oxygen transfer capacities (OTR_{max}). The algebraic sign indicates if the metabolite was formed (positive) or consumed (negative) in the observed cultivation period. In this table, concentrations of oxygen and carbon dioxide are given in g/L and not in mmol/L to be consistent with the concentrations of the other metabolites.

**OTR_{max}**	**Metabolite and cell dry weight formation [g/L]**										
[mmol/L/h]	Glucose	CDW	BDO	Glycerol	Acetoin	Lactate	Ethanol	Succinate	Acetate	Oxygen	CO_2
4.3	-171.3	2.0	70.7	10.6	3.9	1.3	n.d.	n.d.	-0.2	-13.4	82.4
4.4	-163.8	-2.2	69.7	12.2	1.2	-3.5	-0.5	n.d.	n.d.	-11.4	72.3
4.7	-198.1	5.6	86.3	17.8	1.0	2.1	1.4	n.d.	n.d.	-13.3	84.7
5.4	-161.2	5.9	65.0	7.9	1.6	2.0	1.3	n.d.	n.d.	-13.8	91.6
5.7	-196.6	7.0	80.3	12.0	1.3	0.6	1.3	n.d.	n.d.	-13.5	84.3
6.2	-170.8	3.2	68.7	7.8	4.5	1.7	n.d.	n.d.	-0.3	-11.4	66.0
6.4	-161.6	-2.0	68.1	7.2	1.8	-2.4	-0.5	n.d.	n.d.	-13.5	78.2
7.2	-165.6	6.2	67.8	4.2	1.4	5.5	2.9	n.d.	n.d.	-13.7	75.7
9.0	-165.3	6.1	71.0	1.6	2.9	2.6	2.5	n.d.	n.d.	-15.1	77.0
9.2	-169.1	4.9	66.1	2.8	6.0	1.6	n.d.	n.d.	-0.3	-17.1	93.4
9.3	-171.4	-0.7	70.0	0.9	4.9	-7.1	-2.5	n.d.	n.d.	-15.5	82.2
9.4	-181.9	8.0	80.2	0.7	6.4	n.d.	n.d.	n.d.	n.d.	-15.9	80.6
10.1	-163.5	2.6	71.2	1.2	5.0	-5.5	-0.7	n.d.	n.d.	-19.0	87.2
12.7	-161.7	6.6	66.6	n.d.	8.6	-0.9	1.1	n.d.	n.d.	-19.9	80.4
12.8	-188.6	7.4	75.9	n.d.	10.0	n.d.	n.d.	n.d.	n.d.	-20.0	78.7
12.9	-170.1	3.7	61.9	0.4	7.4	-5.8	-2.9	n.d.	n.d.	-20.2	86.7
13.4	-172.9	4.9	60.2	0.2	10.4	-3.1	-2.7	-0.1	n.d.	-23.6	93.8
15.5	-169.3	5.4	55.8	n.d.	16.0	-1.2	-1.6	n.d.	n.d.	-22.4	91.4
16.0	-165.3	-1.2	55.9	n.d.	23.6	n.d.	-0.7	-0.7	n.d.	-26.1	95.5

CDW: Cell dry weight; BDO: 2,3-butanediol; n.d.: not detected

Table S2: Additional calculations to Table S1. This table provides additional information to Fig. 4 and Table S1. The formation rate of mayor metabolites during the 2,3-butanediol production phase (between t_1 and t_2 in Fig. S1) is shown for 19 individual shake flask cultivations with different maximum oxygen transfer capacities (OTR_{max}). The algebraic sign indicates if the metabolite was formed (positive) or consumed (negative) in the observed cultivation period. In this table, formation rates of oxygen and carbon dioxide are given in g/L/h and not in mmol/L/h to be consistent with the formation rates of the other metabolites. Additionally, the balance closures for carbon and degree of reduction balances is presented. A carbon balance closure of 100% indicates that all carbon from the substrate is found in the products. These balances include all compounds listed in Table S1. The average RQ was calculated from the values presented in Table S1 according to Eq. 10.

**OTR_{max}**	**Metabolite formation rate [g/L/h]**						**Balance closure [%]**		**Average RQ [-]**	
[mmol/L/h]	Glucose	BDO	Glycerol	Acetoin	Oxygen	CO_2	Carbon	DoR	calculated	measured
4.3	-1.8	0.7	0.11	0.04	-0.14	0.8	96.4	96.2	4.6	4.1
4.4	-2.0	0.9	0.15	0.01	-0.14	0.9	88.3	89.7	6.0	4.2
4.7	-2.3	1.0	0.20	0.01	-0.15	1.0	100.0	103.6	4.3	4.3
5.4	-2.3	0.9	0.11	0.02	-0.20	1.3	102.5	97.1	5.0	4.4
5.7	-3.0	1.2	0.18	0.02	-0.21	1.3	94.0	95.9	5.1	4.2
6.2	-2.6	1.0	0.12	0.07	-0.17	1.0	89.1	93.1	4.6	3.9
6.4	-2.2	0.9	0.10	0.02	-0.18	1.1	88.8	88.2	5.3	3.9
7.2	-2.8	1.1	0.07	0.02	-0.23	1.3	97.5	99.2	4.0	3.7
9.0	-3.2	1.4	0.03	0.06	-0.30	1.5	98.2	101.2	3.4	3.4
9.2	-3.2	1.3	0.05	0.11	-0.32	1.8	97.7	93.9	4.4	3.7
9.3	-3.3	1.3	0.02	0.09	-0.30	1.6	82.3	81.3	5.6	3.5
9.4	-3.3	1.5	0.01	0.12	-0.29	1.5	97.4	101.5	3.1	3.4
10.1	-2.6	1.1	0.02	0.08	-0.30	1.4	94.5	94.8	4.0	3.1
12.7	-3.3	1.4	0.00	0.18	-0.41	1.7	98.9	102.5	2.7	2.7
12.8	-4.0	1.6	0.00	0.21	-0.43	1.7	92.2	98.2	2.9	2.6
12.9	-3.4	1.2	0.01	0.15	-0.41	1.7	83.7	81.7	4.4	2.9
13.4	-3.0	1.0	0.00	0.18	-0.41	1.6	88.4	86.0	4.2	2.6
15.5	-3.7	1.2	0.00	0.35	-0.50	2.0	92.8	90.7	3.3	2.7
16.0	-3.2	1.1	0.00	0.46	-0.51	1.9	98.7	98.9	2.5	2.4

DoR: Degree of reduction

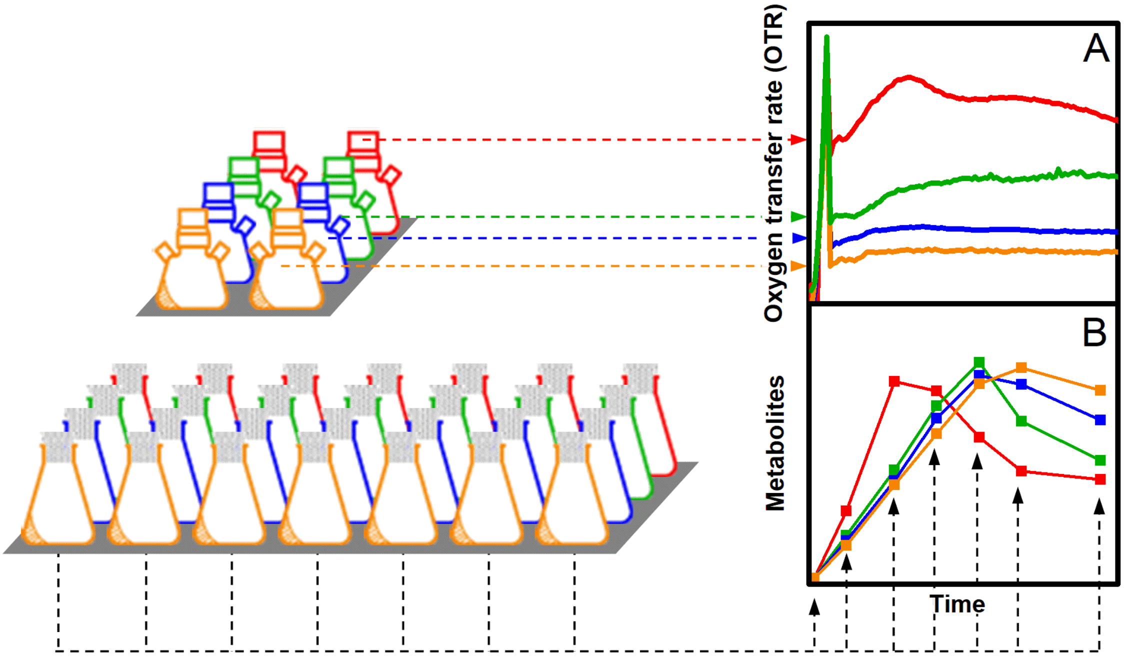

Figure S1: Schematic illustration of the experimental setup of shake flask experiments. Cultivations are performed in multiple individual shake flasks in parallel. As indicated by different colors, four different experimental conditions are investigated in one experiment. Online measurement (A) of oxygen transfer rate (OTR), carbon dioxide transfer rate (CTR) and the respiratory quotient (RQ) is performed as duplicates in a Respiration Activity Monitoring System (RAMOS). Offline samples (B) are taken from individual shake flasks, which are not returned to the shaker after sampling. The figure is adapted from Heyman, et al. [10].

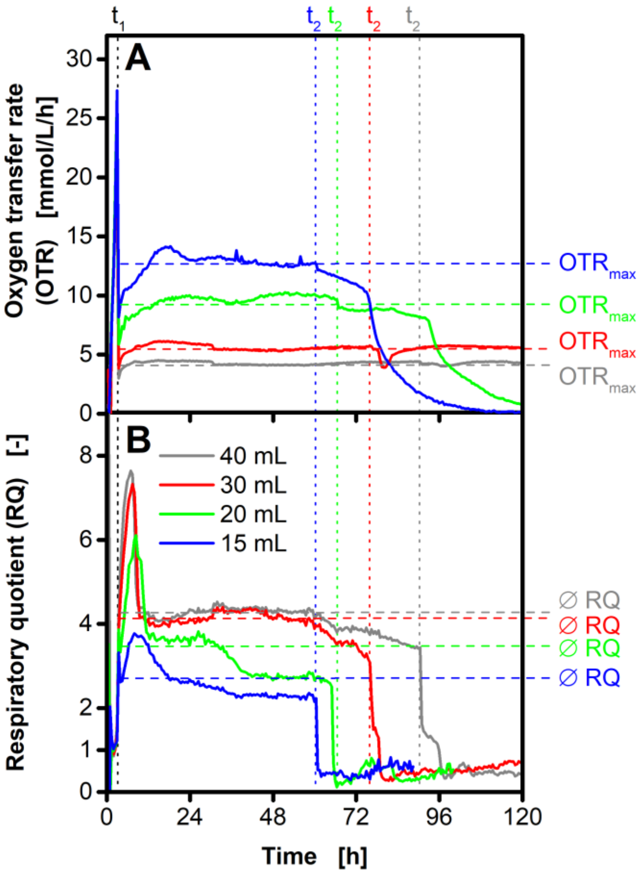

Figure S2: Cultivation of *Bacillus licheniformis* DSM 8785 at different maximum oxygen transfer capacities. The courses of oxygen transfer rates (OTR) (A) and the respiratory quotients (RQ) (B) are shown for cultivations with different filling volumes. The shaking frequency was reduced from 350 to 100 rpm after 3 h (vertical dotted line at t_1). 2,3-Butanediol production begins upon reduction of the shaking frequency (t_1) and ends when the RQ drops (t_2). The average RQ during 2,3-butanediol production (Ø RQ) and the maximum oxygen transfer capacity (OTR_{max}) are depicted. Offline data are derived from an individual shake flask at each time point. Cultivation conditions: 250 mL unbaffled shake flasks, temperature: 37 °C, shaking frequency: 350/100 rpm, shaking diameter: 50 mm.

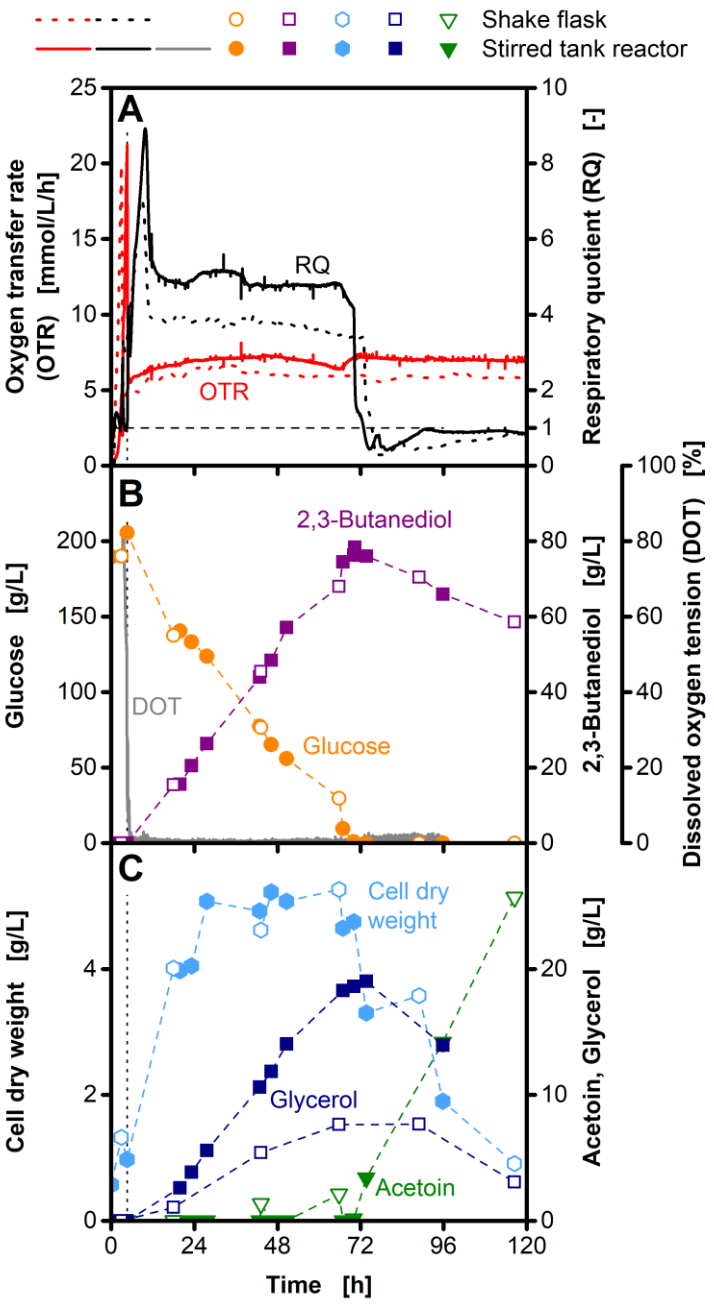

Figure S3: Comparison of shake flask and stirred tank reactor cultivations of *Bacillus licheniformis* DSM 8785. The courses of oxygen transfer rates (OTR) and respiratory quotients (RQ) (A), glucose and 2,3-butanediol concentrations (B) as well as cell dry weight, acetoin and glycerol concentrations (C) are shown. The dissolved oxygen tension (DOT) (B) is only shown for the stirred tank reactor cultivation. To guide the eye, offline samples from shake flask and stirred tank reactor are connected with the same line (except for glycerol). The shaking frequency and the agitation rate were reduced from 350 to 100 and from 1000 to 500 rpm after 4.5 and 3 h, respectively (the vertical dotted line shows 4.5 h). For the shake flask experiment, offline data are derived from an individual shake flask at each time point. For the stirred tank reactor experiment, all samples were taken from one reactor.

Cultivation conditions: Temperature: 37 °C; shake flask cultivation: 250 mL unbaffled shake flasks, filling volume: 30 mL, shaking frequency: 350/100 rpm, shaking diameter: 50 mm; stirred tank reactor cultivation: 2 L stirred tank reactor, filling volume: 1.5 L, aeration rate: 0.5 L/min (0.33 vvm), agitation rate: 1000/500 rpm. The figure is adapted from Heyman, et al. [10].

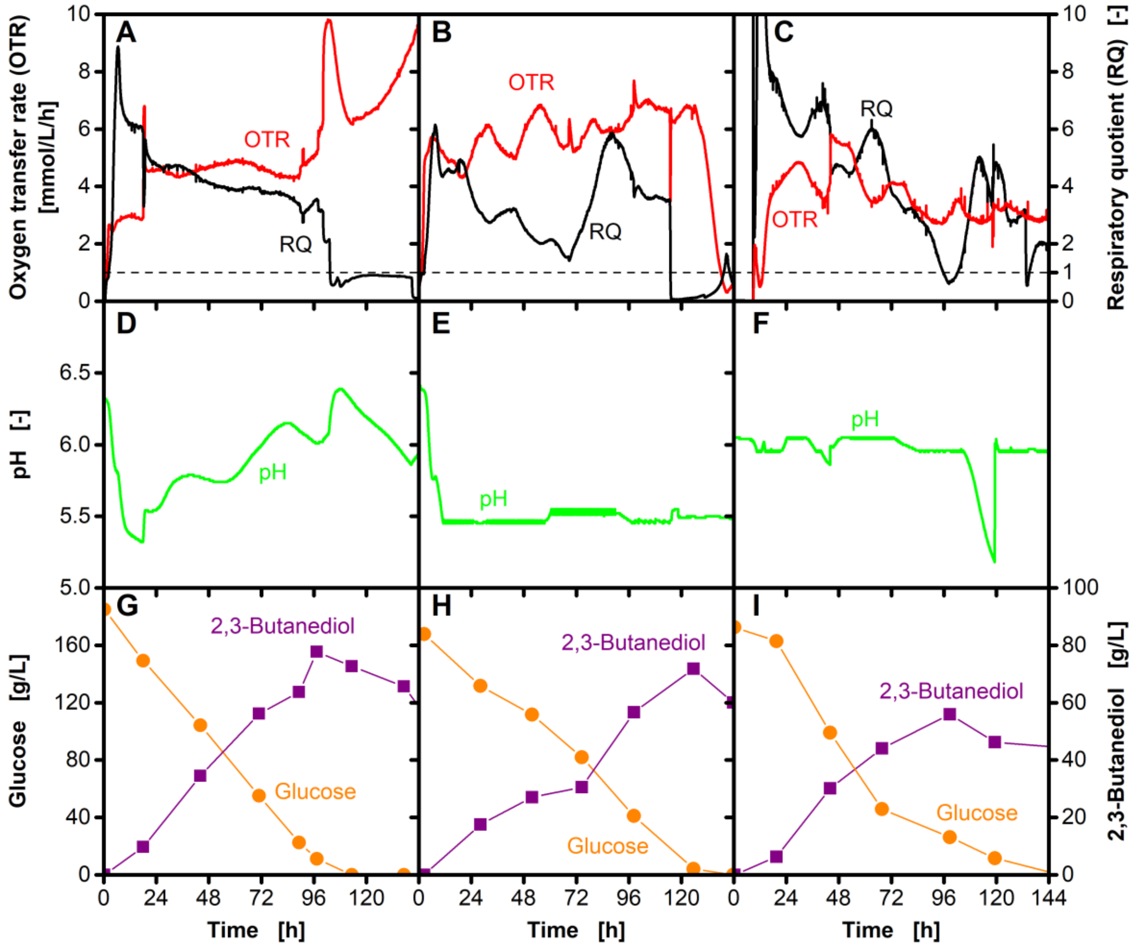

Figure S4: Stirred tank reactor cultivations of *Bacillus licheniformis* DSM 8785 with and without pH control. The courses of oxygen transfer rates (OTR) and respiratory quotients (RQ) (A-C), pH (D-F), as well as glucose and 2,3-butanediol concentrations (G-I) are shown. The cultivations were performed without pH control (A, D, G), or with pH set points of 5.5 (B, E, H) and 6 (C, F, I). The agitation rate was set to 450 rpm in the pH-controlled cultivations, in the cultivation without pH control, the agitation rate was increased from 400 to 450 rpm after 18 h. Cultivation conditions: 2 L stirred tank reactor, temperature: 37 °C, filling volume: 1.5 L, aeration rate: 0.5 L/min (0.33 vvm).

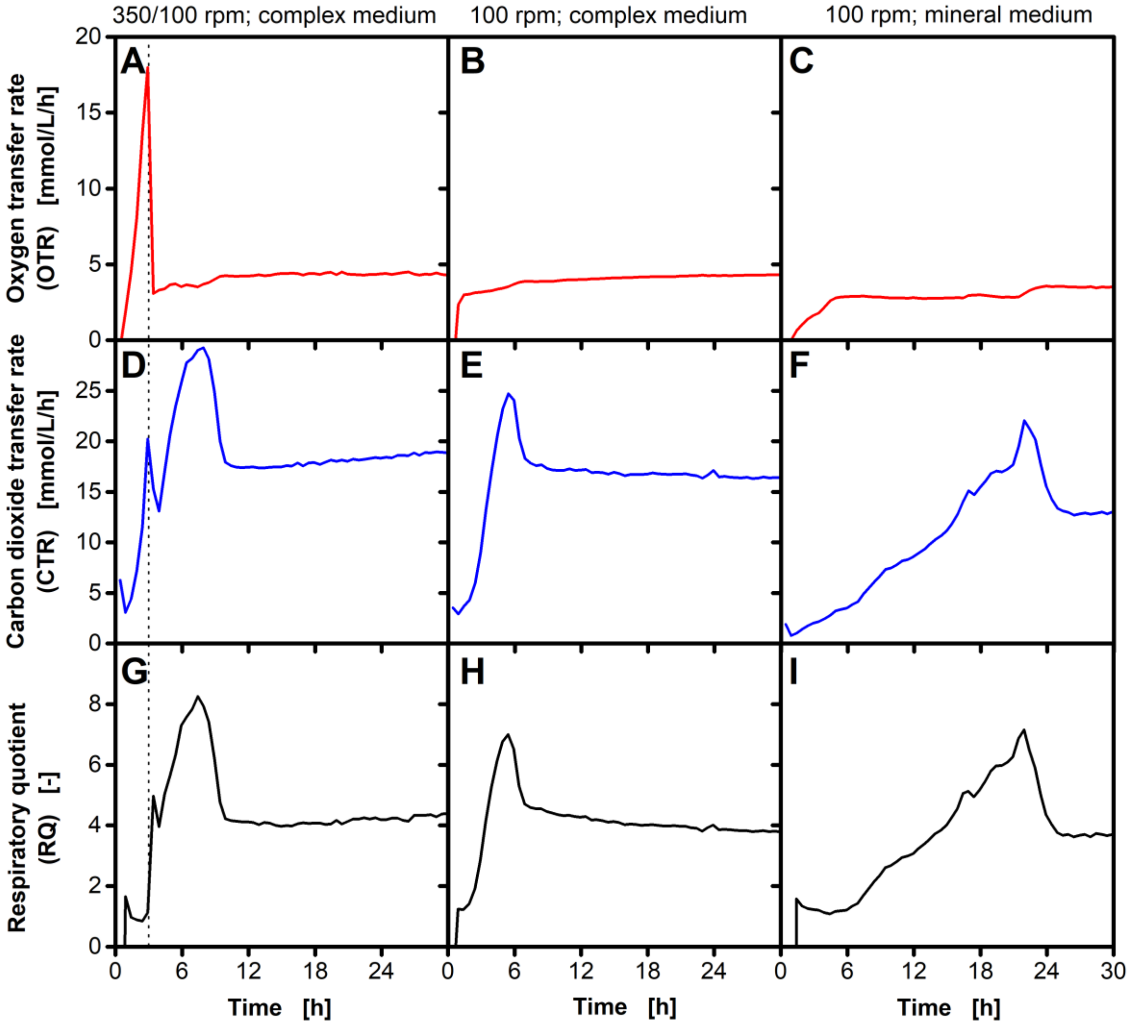

Figure S5: Shake flask cultivations of *Bacillus licheniformis* DSM 8785. The courses of oxygen transfer rates (OTR) (A-C), carbon dioxide transfer rates (CTR) (D-F) and respiratory quotients (RQ) (G-I) are shown. As indicated in the figure, the shaking frequency was either reduced from 350 to 100 rpm after 3 h (vertical dotted line; A, D, G) or kept at 100 rpm throughout the cultivation (B, C, E, F, H, I). Cultivations in complex medium (as described in Section 2.1) and mineral medium are compared. The composition of the mineral medium is: 180 g/L glucose, 7 g/L K_2HPO_4, 5.5 g/L KH_2PO_4, 3.6 g/L NH_4NO_3, 1 g/L $(NH_4)_2SO_4$, 1 g/L $MgSO_4 \cdot 7H_2O$, 0.12 g/L $Na_2MoO_4 \cdot 2H_2O$, 0.026 g/L $CaCl_2 \cdot 2H_2O$, 0.029 g/L $Co(NO_3)_2 \cdot 6H_2O$, 0.07 g/L $(NH_4)_2Fe(SO_4)_2 \cdot 6H_2O$, 0.002 g/L nicotinic acid, 0.0002 g/L Na_2SeO_3, 0.00006 g/L $NiCl_2 \cdot 6H_2O$, 0.05 g/L $MnCl_2 \cdot 4H_2O$, 0.001 g/L H_3BO_3, 0.0002 g/L $AlK(SO_4)_2 \cdot 12H_2O$, 0.00021 g/L $CuCl_2 \cdot 2H_2O$, 0.0003 g/L $ZnCL_2$ and 0.0055 g/L $Na_2EDTA \cdot 2H_2O$ with an initial pH of 6.8. Cultivation conditions: 250 mL unbaffled shake flasks, temperature: 37 °C, filling volume: 40 mL, shaking diameter: 50 mm.

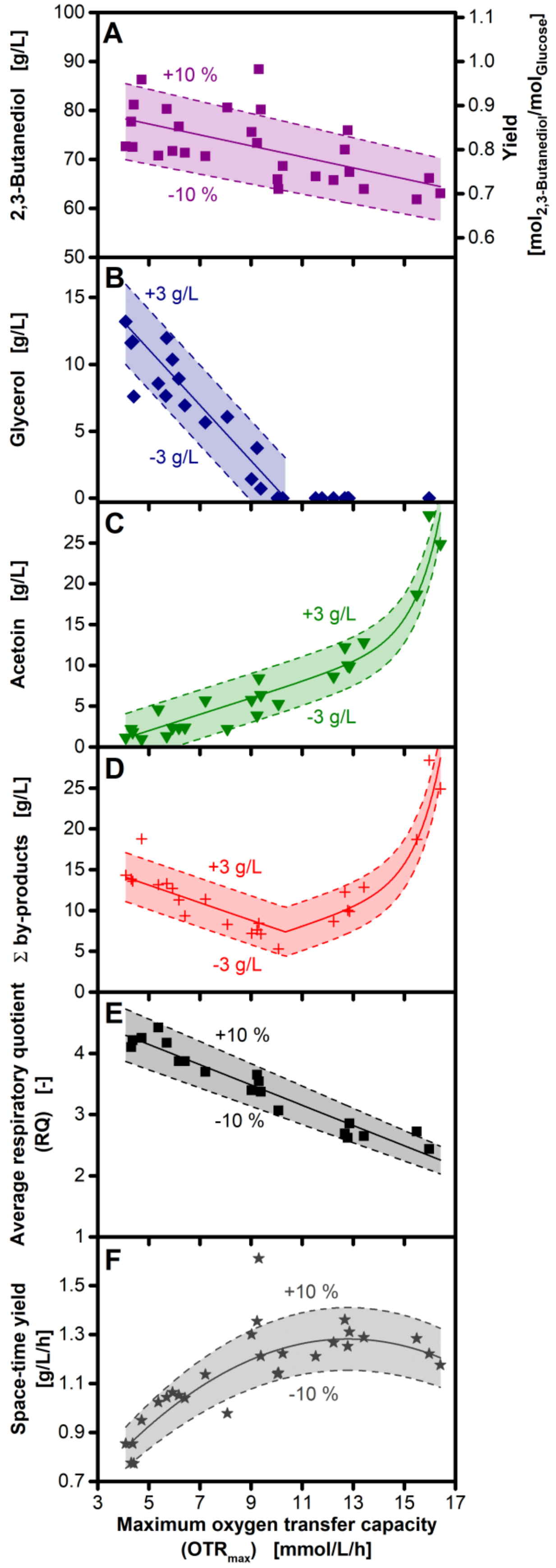

Figure S6: Variation of the maximum oxygen transfer capacity and the average RQ influences product formation during 2,3-butanediol production with *Bacillus licheniformis* DSM 8785. Maximum 2,3-butanediol (A), glycerol (B) and acetoin concentrations (C), the sum of accumulated by-products (D), the average RQ (E) and the space-time yield (F) are shown in dependency of the maximum oxygen transfer capacity (OTR_{max}) during the 2,3-butanediol production phase. The solid lines represent the fitted linear (A, B and E), exponential (C) or cubic (F) correlations. The solid line in D was calculated as sum of the fitted lines from B and C. Error margins (10% for A, D, E and F; 3 g/L for B, C and D) are represented by dashed lines. The figure is adapted from Heyman, et al. [10] and details about the experimental procedure are described in detail in that publication.
